# Alternative Splicing Generates Different 5′ UTRs in OCT4B Variants

**Published:** 2017

**Authors:** Ensieh M. Poursani, Majid Mehravar, Alireza Shahryari, Seyed Javad Mowla, Bahram Mohammad Soltani

**Affiliations:** Department of Molecular Genetics, Faculty of Biological Sciences, Tarbiat Modares University, Tehran, Iran

**Keywords:** Alternative splicing, Genes, 5′ untranslated regions

## Abstract

**Background:**

The human *OCT4* gene, responsible for pluripotency and self-renewal of Embryonic Stem (ES) and Embryonic Carcinoma (EC) cells, can generate several transcripts (OCT4A, OCT4B-variant 2, OCT4B-variant 3, OCT4B-variant 5, OCT4B1, OCT4 B2 and OCT4B3) by alternative splicing and alternative promoters. OCT4A that is responsible for ES and EC cell stemness properties is transcribed from a promoter upstream of Exon1a in those cells. The OCT4B group variants (OCT4B-variant2, OCT4B-variant3, OCT4B-variant5, OCT4B1, OCT4B2 and OCT4B3) are transcribed from a different promoter located in intron 1 and some of them respond to the cell stresses, but cannot sustain the ES/EC cell self-renewal. However, the exact function of OCT4B group variants is still unclear.

**Methods:**

In the present study, we employed RT-PCR and sequencing approaches to explore different forms of *OCT4* transcripts.

**Results:**

Our data revealed that the OCT4B group variants (OCT4B-variant2, OCT4 B-variant3, OCT4B1, OCT4B2 and OCT4B3) have longer 5′ UTR in the human bladder carcinoma cell line of 5637.

**Conclusion:**

These *OCT4* variants undergo alternative splicing in their 5′ UTR which might exert regulatory roles in transcription and translation mechanisms.

## Introduction

The *OCT4*, an important transcription factor responsible for stemness, is a main regulator of pluripotency and self-renewal retaining in the Embryonic Stem (ES) and Embryonic Carcinoma (EC) cells ^1–3^. This gene encodes for two types of variants; OCT4A and OCT4B group variants (OCT4B-variant 2, OCT4B-variant 3, OCT4B-variant 5, OCT4B1, OCT4B2 and OCT4B3) ^4^. OCT4A is known to induce stemness properties in ES and EC cells and in contrast, OCT4B variants cannot sustain self-renewal and pluripotency of embryonic stem cells. Some studies showed that OCT4B variants (OCT4B-190 and OCT4B-265 isoforms) are up-regulated during cell stresses ^5^,^6^. OCT4B1 is highly expressed in undifferentiated cells and down-regulate during cell differentiation ^7^. OCT4B group variants are produced by alternative promoter and alternative splicing events in various human cancer cell lines. Since these *OCT4* variants seem to be important in cancer cells and also, most of them generate the same protein isoforms, it was decided to evaluate 5′-UTR sequences of known OCT4B group variants in bladder cancer cell line of 5637 that expresses most of *OCT4* transcripts.

The 5′ Untranslated Region (5′ UTR) is located upstream of the start codon (AUG), and plays crucial roles in regulating transcription and translation of related mRNAs ^8^. The whole or partial sequence of 5′ UTR, which is called uORF (upstream Open Reading Frame), can be translated into single or several short peptides that can regulate translation of the main coding sequence of the same mRNA. Also, the existence of unique regulatory elements in 5′ UTR might influence the protein translation by providing binding sites for regulatory factors ^9^. In this study, the length and structure of the 5′ UTRs of OCT4B group variants were investigated by *in silico* analysis and experimental approaches such as RT-PCR and sequencing in the human bladder cancer cell line of 5637.

## Materials and Methods

### Cell culture, RNA extraction and cDNA synthesis

5637 cells were cultured in RPMI-1640 supplemented with 10% FBS and 1% penicillin/streptomycin and incubated at 37*°C* and 5% CO_2_. Then, 5637 cells were harvested and the RNA was extracted using TRIzol reagent according to the instruction of manufacturer. Quality and quantity of extracted RNA was evaluated by agarose gel electrophoresis and spectrophotometry, respectively. Complementary DNA (cDNA) was synthesized using 2 *μg* RNA with reverse transcriptase enzyme, according to the manufacturer’s instruction (Fermentase).

### Reverse Transcription (RT)-Polymerase Chain Reaction (PCR)

RT-PCR was performed by F/R primer set using amplicon Taq DNA polymerase. The primer sequences were as follows: F; 5′-AGGGCTCTTTGTCCACTTTGTATAG-3, R; 5′-CTCAAAGCGGCAGATGGTCG-3′; GAPF (5′-GC CACATCGCTCAGACAC-3′) and GAPR (5′-GGCAA CAATATCCACTTTACCAG-3′). PCR program was carried out at 94°*C* for 4 *min*, amplification for 35 cycles with denaturation at 94°*C* for 30 *s*, annealing at 65°*C* for 30 *s* and extension at 72°*C* for 30 *s*, with a final extension at 72°*C* for 7 *min*. The thermal profile for GAPDH was performed for 28 cycles with annealing at 58°*C* for 30 *s* and extension at 72°*C* for 15 *s*.

### DNA cloning and sequencing

PCR products were excited from agarose gel, purified by DNA purification kit (GeneAll Biotechnology, South Korea) and cloned into the pTZ57R/T vector. Positive colonies containing recombinant vectors were selected by colony PCR. Recombinant vectors were extracted by plasmid extraction kit (GeneAll Biotechnology, South Korea) and sequenced (Applied Biosystems, South Korea).

## Results

### B group variants of OCT4 have longer 5′ UTRs

**Figure 1.**
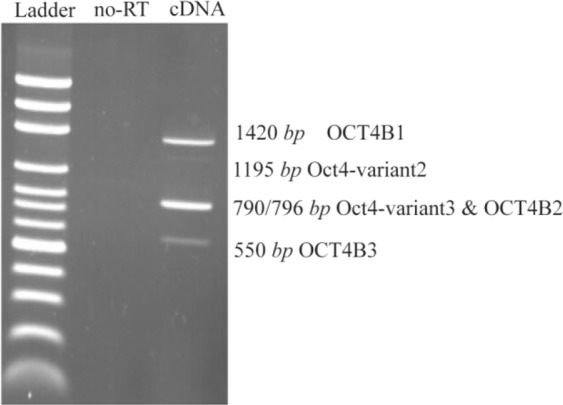
Reverse Transcription (RT)-Polymerase Chain Reaction (PCR). RT-PCR was performed using primer set F/R for 5637 cells. The predicted PCR product sizes were 1420, 1195, 790/796 and 550-bp corresponded for OCT4B1, OCT4B-variant2, OCT4B-variant3/OCT4B2 and OCT4B3, respectively. Ladder is DNA marker 100-bp, and no-RT sample was used as a control to confirm absence of DNA contaminations.

RT-PCR assay was performed using a new forward primer located upstream of 5′ UTR of the OCT4B group variants and PCR products were cloned into PTZ57R/T vector and sequenced. The sequencing results were blasted in NCBI database against the human genome and transcriptome and it was indicated that OCT4B group variants have a longer 5′-UTR with different sequences. Based on our sequencing data, the OCT4B group 5′ UTRs were 88-*bp* longer than those which have been registered in NCBI databank (Figure 1).

### Alternative splicing generates different 5′ UTRs in OCT4B group variants

**Figure 2.**
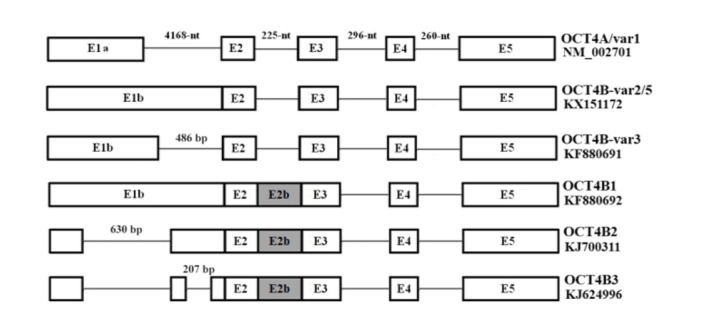
A schematic structure of *OCT4* variants. OCT4A is composed of 5 exons which exon 1a is specific for this variant. Other OCT4B group variants have exon 1b instead of exon 1a and have longer 5′ UTR. Also, this figure indicates occurring alternative splicing in 5′ UTR of OCT4B group variants, specifically in exon 1b. It should be mentioned that OCT4B-variant 2 and OCT4B-variant 5 are different just in one SNP that cause generating longer isoform in OCT4B-variant 5.

**Figure 3.**
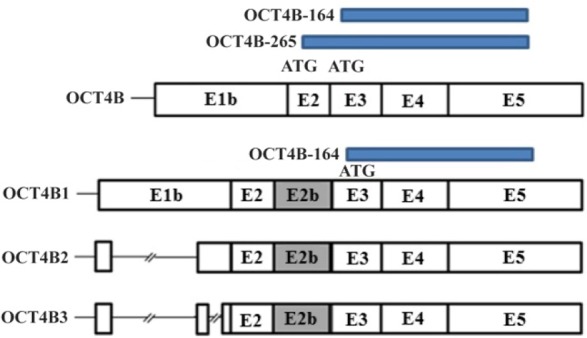
Protein isoforms produced by *OCT4* transcripts. OCT4B transcript can produce two isoforms that initiate from ATG start codon (OCT4B-265 and OCT4B-164) and one isoform initiates from CTG start codon (OCT4B-190). OCT4B1, OCT4B2 and OCT4B3 can potentially translate to a 164 amino acid protein that is the same with OCT4B-164 isoform

Alignment of sequencing data with online tools such as NCBI BLAST, GeneBee multiple alignment and CLUSTALW showed that the 5′ UTR of the OCT4B group transcripts undergo alternative splicing in various *OCT4* variants. OCT4B1 had a complete 5′ UTR, but OCT4B, OCT4B2 and OCT4B3 lost some parts of their 5′ UTR by splicing (Figure 2). However, alternative splicing in 5′ UTR sequences of OCT4B group variants does not affect amino acid sequence of their proteins; it might influence the transcription and translation efficiencies (Figure 3).

### In silico analysis of OCT4 5′UTR sequences

Based on uORF theory within 5′-UTR of mRNAs which translate to small peptides and can regulate eukaryotic gene expression), deleted sequences of *OCT4* 5′UTRs were translated using expasy translate tool. Our *in silico* analysis revealed that deleted region of 5′UTRs in the *OCT4*-variant3, OCT4B2 and OCT4B3 can produce 6, 6 and 4 peptides potentially. The peptides resulted from *OCT4*-variant 3 5′-UTR have 5, 9, 11, 14, 15 and 30 residues length. Also, OCT4B2 5′-UTR can potentially be translated into peptides with 5, 7, 8, 9, 45 and 49 residues. Moreover, OCT4B3 5′-UTR can potentially be translated into 3 peptides with 5, 15 and 30 residues.

## Discussion

*OCT4* is a crucial and dependent determinant of pluripotency in ES and EC cells ^10^. The regulatory role of *OCT4* in pluripotency and self-renewal in ES and EC cells introduced this gene as a pluripotency marker ^11^. This gene has two potential promoters that generate OCT4A and OCT4B group variants (OCT4B-variant2, OCT4B-variant3, OCT4B-variant5, OCT4B1, OCT4 B2 and OCT4B3) ^12^. OCT4A expression is normally attributed to pluripotent cells such as primordial germ cells (PGCs), ES and EC cells ^10^,^13^.

The OCT4B group variants are expressed in different cancer cells, differentiated cells and undifferentiated cell types. While some studies proposed the role of these variants in response to the cell stresses, little information exists about the exact expression, function and structure of these variants ^1^,^5^.

When investigating on the new variants of *OCT4* gene, it was revealed that OCT4B group variants have longer 5′ UTR and their 5′ UTRs have different sequences due to alternative splicing (GenBank accession numbers: KF880691 for OCT4B-variant3, KF880692 for OCT4B1, KJ700311 for OCT4B2, KJ624996 for OCT4B3 and KX151172 for *OCT4*-variant 2).

Longer and various 5′ UTR, respectively provides more and different regulatory elements that might influence the efficiency of transcription, translation and even function of protein products of a single gene.

In most of past studies, 3′ UTRs were considered as important targets for gene regulation. For example, microRNAs could target 3′ UTR sequences of various genes and down-regulate gene expression via translation inhibition. Some microRNAs such as miR-145 and miR-302a can inhibit *OCT4* translation and regulate *OCT4* gene expression during development, respectively ^14^,^15^. However, there is no comprehensive study for the importance of 5′ UTR sequence on the *OCT4* gene expression during transcription and translation mechanisms.

All over, alternative splicing in 5′UTR sequence and using various 5′UTR for several products of the same gene can play regulatory roles for translation efficiencies under different conditions, also in various tissues, during different stages of development and even in various cancers and diseases. It is supposed that OCT4B group protein levels might be regulated by using different 5′ UTR sequences in OCT4B group variants during various human cancers and diseases.

## Conclusion

Therefore, it is hoped that further studies such as cloning of various 5′-UTR sequences in upstream, a reporter gene and evaluating the translation rate of that reporter gene will help to better understand the diversity in 5′UTR and its effect on the translation efficiency and function of each variant.
